# Altered expression of genes related to innate antifungal immunity in the absence of galectin-3

**DOI:** 10.1080/21505594.2021.1903212

**Published:** 2021-03-29

**Authors:** Caroline Patini Rezende, Patricia Kellen Martins Oliveira Brito, Andre Moreira Pessoni, Thiago Aparecido Da Silva, Gustavo H Goldman, Fausto Almeida

**Affiliations:** a Department of Biochemistry and Immunology, Ribeirao Preto Medical School, University of Sao Paulo, Ribeirao Preto, SP, Brazil; b Department of Cellular and Molecular Biology, Ribeirao Preto Medical School, University of Sao Paulo, Ribeirao Preto, SP, Brazil; c Departamento De Ciencias Farmaceuticas, Faculdade De Ciencias Farmaceuticas De Ribeirao Preto, Universidade De Sao Paulo, Ribeirao Preto, SP, Brazil

**Keywords:** Galectin-3, gene knockout, innate immune response, antifungal immunity, gene expression

## Abstract

Galectin-3 (Gal-3) is the most studied member of the animal galectin family, which comprises β-galactoside-binding lectins and participates in several cellular events. Its expression in cells involved in innate and adaptive immunity is related to anti- and proinflammatory functions, signaling an important role in inflammatory, infectious, and tumorigenesis processes. Mice deficient in Gal-3 exhibit important phenotypes, but it is unclear whether these phenotypes reflect an impairment of the functions of this protein. Gal-3 plays an important role in modulating the immune response to different pathogenic microorganisms. However, the role of Gal-3 in immunity to infection is still poorly understood. Therefore, we investigated the effects of Gal-3 deletion on the expression of genes involved in the innate immune response in the lungs, spleens, and brains of Gal-3 KO mice. Gene profiling expression analysis suggested that Gal-3 deletion resulted in differentially modulated expression of the genes encoding beta-glucan, mannose and chitin-responsive pattern recognition receptors, signal transduction, inflammation, and phagocytosis. Our data thus suggest the importance of Gal-3 expression in the host innate immune system.

## Introduction

Galectins are a family of animal β-galactoside-binding lectins [1,^2^]. This family consists of 15 multifunctional members [3], which are expressed in the nucleus, cytoplasm or cell membranes, or secreted extracellularly [1]. Galectins can bind glycoconjugates on the cell surface, triggering a transmembrane signaling cascade [3–5], which modulates several biological processes such as apoptosis, immune cell activation, cell adhesion, and cytokine secretion [6].

Galectin-3 (Gal-3) is the most studied member of the galectin family. The protein is expressed in various types of immune cells, tissues, and organs at different stages of development [7,8]. Its expression depends on the stage of the cell cycle and the metabolic status of the cell [9,10], while its biological functions are defined by its intracellular selectivity or extracellular localization [11]. Gal-3 can associate with the cell surface or localize in the extracellular matrix [12,13]. Extracellularly, Gal-3 exerts several autocrine and paracrine effects, which can mediate cell adhesion and activation by acting as chemoattractants for certain types of cells [3].

The expression of Gal-3 in cells of the innate and adaptive immune systems is related to anti- and proinflammatory functions [14–17], signaling important roles in inflammatory and infectious processes [16]. Depending on their ligands, Gal-3 has varied functions and participates in several cellular events [16]. Therefore, Gal-3 pathophysiological activities are associated with autoimmune diseases, tumorigenesis, and fungal or parasitic infections [18].

Notably, mice deficient in Gal-3 exhibit several important phenotypes such as impaired development [19], inflammatory functions [20] and stress control increased compulsive behavior [21] and high susceptibility to fungal infections [22–25]. Although it is unclear whether these phenotypes reflect an impairment of the intra- or extracellular functions of this protein [26]. In the population, the existence of single nucleotide polymorphisms (SNPs) in the gene encoding Gal-3 (*lgals3*) promotes variations in their serum levels in children with respiratory tract infections [27] and in diseases such as rheumatoid arthritis [28], malignant tumors [29], cardiomyopathies [30], but there is nothing related to fungal infections. Studies show that SNPs in the *ptx3* [31] and *clec7a* [32] are associated with an increased risk of developing aspergillosis and candidiasis. Also, the presence of SNPs in host immunity genes, especially in standard recognition receptors (PRRs) such as toll-like receptors [33], C-type lectin receptors [34,35], pentraxins [36] and tumor necrosis factor receptors [37,38], may contribute to agreater susceptibility to fungal infections [39].

Therefore, we investigated whether Gal-3 deficiency promoted changes in the expression of innate immune response in the lungs, spleens and brains of Gal-3 KO mice compared to wild-type controls. Gene expression profiling analysis suggested that the absence of Gal-3 was associated with both enhanced and decreased expression of genes involved in the transcription of beta-glucan, mannose and chitin-responsive pattern recognition receptors (PRRs), signal transduction, inflammation, and phagocytosis. Our data suggest the importance of Gal-3 expression during innate immune responses.

## Materials and methods

### Animals

Six-to-eight-week-old, male C57BL/6 (wild type, WT) and Gal-3 Knockout (Gal-3 KO) mice were obtained from the animal housing facility at the Ribeirao Preto campus at the University of São Paulo (Ribeirao Preto, São Paulo, Brazil). Gal-3 KO mice were generated in the C57BL/6 mouse background as previously described and bred for nine generations [20]. The animals were housed in the animal facility at Ribeirao Preto Medical School at the University of São Paulo under optimized hygienic conditions. All animal experiments were conducted according to the Brazilian College of Animal Experimentation Protocol 100/2015 and approved by the Committee on Ethics in Animal Research at Ribeirao Preto Medical School at the University of São Paulo.

### PCR array

WT and Gal-3 KO mice were euthanized and lung, spleen and brain fragments were aseptically harvested and homogenized in a tissue homogenizer (IKA® T10 basic homogenizer). Total RNA was extracted from the lung, spleen and brain homogenates using TRIzol reagent (Invitrogen Corporation, California, USA), according to the manufacturer’s instructions. The concentration of the eluted total RNA was assessed by absorbance ratios (A260/A280 and A260/A230) on a spectrophotometer (NanoDrop, Thermo Fisher Scientific, Wilmington, Delaware, USA). RNA integrity was evaluated by electrophoresis on a 1.8% agarose gel to check the presence of the 28S and 18S bands. cDNA was synthesized by reverse transcription with oligo d(T) primers using the ImProm-II^TM^ Reverse Transcription System Kit (Promega Corp., Fitchburg, WI). The Mouse Antifungal Response RT^2^ Profiler PCR Array Kit (Qiagen Cat# PAMM-147Z) was used for gene expression analysis. This arrangement consisted of a plate containing 84 lyophilized primers targeting genes involved in the innate immune response to fungal infection (cited in Table S1), and negative controls for genomic DNA and primers targeting six different constituent genes. Pooled cDNA was mixed with buffer, DNA polymerase, SYBR Green Mastermix (2X RT^2^ SYBR Green Mastermix, SABiosciences-Qiagen), and water and partitioned among the 96 wells of the plate (25 μL per well). Real-time PCR was performed in a BioRad CFX 96 thermal cycler (C1000 ^TM^ thermal cycler) with the following cycling conditions: 95°C for 10 min; then, 40 cycles of 95°C for 15 sec and 60°C for 60 sec. Ct (threshold cycle) values were analyzed using SABiosciences Web software available online through the Qiagen Data Analysis Center website.

### Statistical analysis

The Ct values were normalized using selected reference genes (*gusb, hprt1, hsp90ab1, gadph* and *actb*), which showed more stable expression between experimental and control groups. If more than one control gene was selected for normalization, the geometric mean of its values was performed. Our data were normalized with *b2m, gapdh, gusb*, and *hsp90ab1* for the lungs; *hsp90ab1* for the spleens, and *actb, b2m*, and *gusb* for the brains. The Ct values were geometrically calculated and used to determine 2^−ΔΔCt^. Differences in transcript levels (fold change (FC)) between experimental and control groups were determined using the Ct comparison method, based on the 2^−ΔΔCt^ algorithm. Genes were considered significantly modulated (induced or repressed) if the difference in the mean 2^−ΔΔCt^ values was greater than 2 or less than −2.

## Results

### Genes upregulated in Gal-3 KO mice compared to wild-type mice

**Figure 1.**
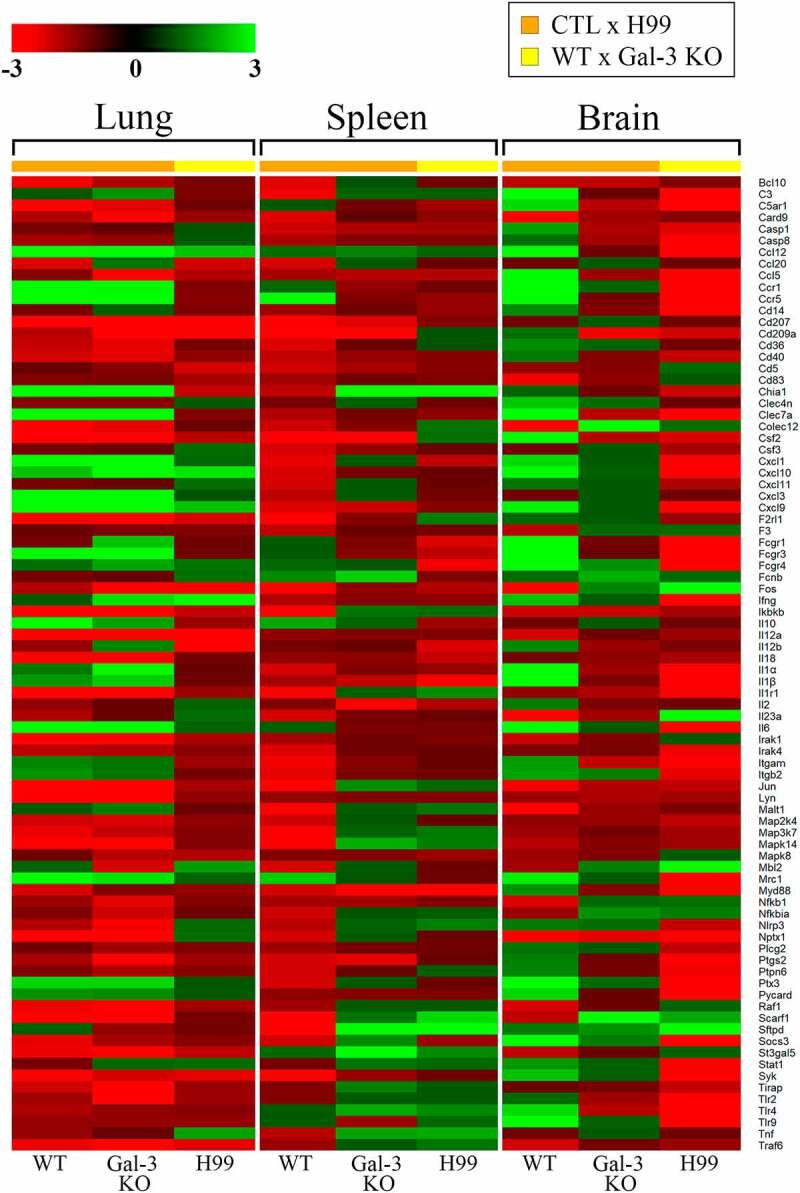
Heat map showing expression of genes in the innate immune response in the absence of Gal-3

**Figure 2.**
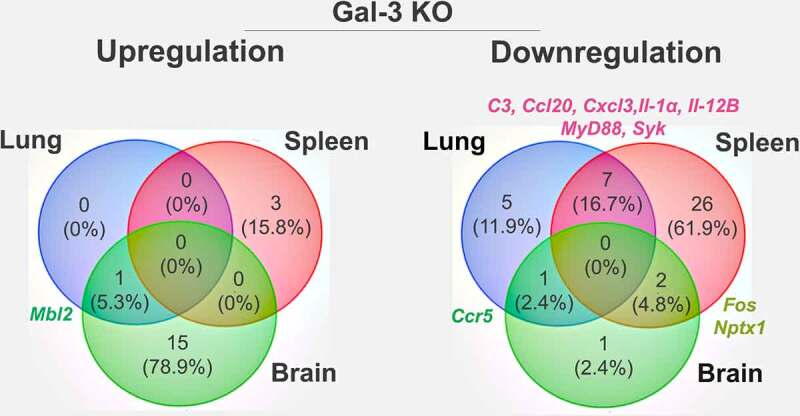
Up- and downregulation of innate immunity genes in Gal-3 KO mice

The levels of expression of 84 genes present in the Mouse Antifungal Response RT^2^ Profiler PCR Array Kit were evaluated in Gal-3 KO mice relative to WT mice. This approach helped elucidate the role of Gal-3 in modulating genes expressed during the innate immune response (Figure 1 and Table S2). A total of 19 genes were upregulated in the lungs, spleens and brains of Gal-3 KO mice, with fold regulation greater than 2. Notably, only mannose-binding lectin 2 (*mbl2*), which is involved in the initial activation of the complement system, inflammation and phagocytosis, was overexpressed in the lungs (Table S3). In the spleen, genes encoding the chemokine (C-C-motif) receptor 5 (*ccr5*), mannose receptor C type 1 (*mrc1*), and toll-like receptor 9 (*tlr9*) were upregulated (Table S4). Sixteen genes were upregulated in the brain, including complement component 3 (*c3*), caspase 1 (*casp1*), chemokines (*ccl5, ccl12, cxcl10), cd36, cd209a*, C-type lectin domain family 4 and 7 (*clec4n* and *clec7a*), colony stimulating factor 2 (*csf2*), interleukins (*il-1β, il-2, il-12b*), mannose-binding lectin 2 (*mbl2*), pentraxin (*ptx3*), and surfactant-associated protein D(*sfpd*) (Table S5). These gene expression profiles demonstrated that absence of Gal-3 upregulates pro-inflammatory mediators and PRRs, with the notable highlight of *mbl2* upregulation in the lungs and brain (Figure 2).

Gene expression analyses in the lungs, spleens and brains of Gal-3 KO and WT mice (control) during the innate response. Ct (threshold cycle) values were transferred to SABiosciences Web software available online via the Qiagen website. Fold regulation cut off = 2 and pvalue cut off = 0,05. The scale represents fold change, with threshold set to 3 (green: upregulated, red: downregulated, black: medium change).

Comparative Venn diagram showing genes up- and downregulated in the lungs, spleens and brains of Gal-3 KO mice. Mbl2 and Ccr5 were up- and downregulated, respectively, in the lungs and brains. C3, Ccl20, Cxcl3, Il-1α, Il-12b, Myd88, and Syk were negatively modulated in spleens and lungs. Fos and Nptx1 were downregulated in spleens and brains. Venn diagram was designed using Venny 2.1.

### Genes downregulated in Gal-3 KO mice compared to wild-type mice

The absence of Gal-3 in downregulating genes expressed in innate antifungal immunity in the lungs, spleens, and brains of Gal-3 KO mice was evaluated. Of the 84 genes tested, 42 genes were downregulated in the three types of tissues studied, with fold regulation less than −2 (Figure 1 and Table S2). In the lungs, 13 genes were downregulated in Gal-3 KO mice, including *c3, ccl20, cxcl3, cxcl10, ccr5, csf2, fcgr1, il-1α, il-6, il-12a, il-12b, myd88*, and *syk*. These genes encode components of the complement system, proinflammatory mediators, chemokines and its receptors, adaptor proteins and effectors of the PRR signal transduction pathway and phagocytosis (Table S3). In the spleens of Gal-3 KO mice, 35 genes were downregulated. These genes were categorized into PRRs (*cd36, cd207, chia1, clec7a, nptx1, ptx3, scarf1*); signal transduction (*bcl10, card9, fos, irak4, ikbkb, itgb2, jun, malt1, map2k4, map3k7, mapk14, syk*); inflammation (*c3, ccl20, cxcll, cxcl3, cxcl11, f3, il-1α, il-1β, il-12b, il1r1, mbl2, myd88*); gene responsive to pathogenic fungi (*socs3*); and phagocytosis (*cd14, fcgr4, sftpd*) (Table S4). Four genes (*ccr5, colec12, fos* and *nptx1*) were downregulated in Gal-3 KO mice brains. *colec12* and *nptx1* are involved in recognition; *fos*, in the signal transduction pathways of toll-like receptors; *ccr5* and *fos*, in inflammation; and *colec12*, in phagocytosis (Table S5). Overall, most of the negatively regulated genes were found in the spleen, while *fos* and *nptx1* were also downregulated in the brain. In addition, seven genes (*c3, ccl20, cxcl3, il-1α, il-12b, myd88, syk*), which were downregulated in Gal-3 KO mice spleens, were also downregulated in the lungs. Only the gene encoding chemokine (C-Cmotif) receptor 5 (*ccr5*) was downregulated in both lungs and brains (Figure 2).

## Discussion

Gal-3 is involved in several biological processes such as angiogenesis, tumor invasion, metastasis, immune response, and maintenance of cellular homeostasis [3,13,40]. This protein is expressed in immune system cells including monocytes, macrophages, B and T lymphocytes, dendritic cells, eosinophils, and neutrophils [41,42], indicating it plays important roles in regulating the host immune response in physiological and pathological conditions [18]. In fungal infections like aspergillosis [43], candidiasis [24], cryptococcosis [22], paracoccidioidomicoses [23,25] and histoplasmosis [17] the influence of Gal-3 was analyzed through the main organs affected by these pathogens such as lungs, spleens and brains. In this study, we conducted expression profiling of genes involved in the host innate immune system in lungs, spleens and brains of Gal-3 KO mice. Our results showed that the absence of Gal-3 promotes both positive and negative modulation of genes encoding PRRs that bind beta-glucan, mannose and chitin, and genes involved in signal transduction, inflammation and phagocytosis. In the lungs and spleen, these genes were preferentially negatively modulated, while in the brain genes were predominantly positively modulated.

The importance of lectins during the innate immune response has been gaining prominence [44]. The antifungal immune response involves fungal recognition by pattern recognition receptors such as C-type lectin receptors (CLRs) [45,46] will mediate the host immune response through opsonization, complement system activation, phagocytosis, and inflammation [15,47]. Also, collectins, pentraxins and complement system proteins can facilitate the engulfment of pathogens by acting as opsonins, as well as promoting direct fungicidal effects [48].

The recognition of fungal pathogens by Gal-3 can be performed in conjunction with Dectin-1, PRR responsible for the recognition of beta glucan, in which macrophages expressing these two lectins are able to produce TNF-α in response to pathogens such as *Saccharomyces cerevisiae* and *Candida albicans* [49]. Moreover, Gal-3 can promote killing of *C.albicans* by binding to beta-1,2-linked oligomannans on the cell surface [50]. Our results suggest that Gal-3 KO promotes both reduced and enhanced expression of genes involved in pathogen recognition in the absence of apathogenic stimulus. This could have implications on the immune response following a fungal infection because Gal-3, as well as other members of the galectin family, they can act as PRRs capable of discriminating the glycans present in the pathogen and host, playing an important role in immune defense [51,52].

Gal-3 may also participate in the phagocytosis of microorganisms by macrophages, triggering activation of the immune system *in vitro* [53,54]. Corroborating our results that showed the decrease in the expression of genes involved in phagocytosis, Sano et al., have verified that Gal-3 KO macrophages showed delayed phagocytosis compared to WT [55]. The addition of exogenous Gal-3 can promote the activation of macrophages in a manner dependent on the properties of this lectin. The hypothesis for the role of Gal-3 in phagocytosis would be that Gal-3 binds to the pathogen and promotes the engulfment by macrophages [55]

C-type lectins such as the mannose-binding protein (Mbl2) and galectins may play a fundamental role in neutralizing the pathogen and conducting adaptive immunity [56]. Our results demonstrated that Mbl2, which can bind to different sugars, including GlcNAc, mannose, fucose and glucose [57], was overexpressed in lungs of Gal-3 KO compared to WT animals. The relationship between Gal-3 and Mbl2 is still unknown, but their interaction may promote several effector functions in the immune system [58].

Gal-3 deletion decreased the expression of chemokines and their receptors (*ccl20, cxcl1, cxcl3, cxcl10, cxcl11, ccr5*), as well as other molecules involved in inflammatory processes (*c3, csf2, il-1α, il-1β, il-6, il-12*) in the lungs and spleen. In agreement with our results, *in vitro* and *in vivo* studies suggest that Gal-3 is important for modulating inflammatory responses due to its roles in cell activation and migration or inhibition of apoptosis [6]. These observations are also supported by studies showing increased expression of galectins and chemokines in inflammatory conditions such as atherosclerosis and osteoarthritis [59–61]. Thus, our results reinforce that Gal-3 is a multifunctional protein involved in several biological events and may play crucial roles in gene expression during the host innate immune response.

## Conclusion

Our results suggested that Gal-3 deletion has implications on the expression of genes involved in PRRs, signal transduction, inflammation and phagocytosis. We hypothesized that Gal-3 may also exert its effects by modulating host immunity. However, additional studies are required to better understand the mechanisms involved and identify whether Gal-3 is an attractive target for the development of therapeutic strategies to enhance antifungal immunity.

## Supplementary Material

Supplemental Material
