# Supplementary material for: Altered expression of genes related to innate antifungal immunity in the absence of galectin-3
Source: Virulence. 2021 Mar 29;12(1):981–8. doi: 10.1080/21505594.2021.1903212 (PMC8009118; doi:10.1080/21505594.2021.1903212)
Supplement: Supplemental Material [file KVIR_A_1903212_SM1221.zip › Document.rtf]

Supplementary Materials: 
Table S1: Genes analyzed using The Mouse Antifungal Response RT² Profiler PCR Array Kit (Qiagen Cat# PAMM-147Z). 
Table S2: Fold Regulation RT² Profiler™ PCR Array Mouse Antifungal Response. 
Table S3: Gene expression profile in the lungs of Gal-3 KO mice compared to WT mice.
 Table S4: Gene expression profile in the spleens of Gal-3 KO mice compared to WT mice. 
Table S5: Gene expression profile in brains of Gal-3 KO mice compared to WT mice. 
